# Supplementary material for: A three-dimensional ratiometric sensing strategy on unimolecular fluorescence–thermally activated delayed fluorescence dual emission
Source: Nat Commun. 2019 Feb 13;10:731. doi: 10.1038/s41467-019-08684-2 (PMC6374486; doi:10.1038/s41467-019-08684-2)
Supplement: Supplementary file 1 — Supplementary Information [file 41467_2019_8684_MOESM1_ESM.pdf]

SUPPLEMENTARY INFORMATION

A Three-Dimensional Ratiometric Sensing Method Based on Unimolecular Fluorescence–Thermally Activated Delayed Fluorescence Dual Emission

Li et al.

Supplementary Information

Supplementary Figure 1. Synthetic routes for compounds 1, 2 and 3.

Supplementary Figure 2. Emission spectra in various solvents.

Supplementary Figure 3. Absorption and Excitation spectra of 3.

Supplementary Figure 4. Linear-fitting Correlation.

Supplementary Figure 5. Emission and lifetime in different solvents at 77K.

Supplementary Figure 6. Photophysical properties in ethanol with different glycterol concentrations.

Supplementary Figure 7. Emission spectra in oxygen free and saturated air condition.

Supplementary Figure 8. Emission spectra of 3 in the complex PLs systems.

Supplementary Figure 9. Photoluminescence lifetime of 3 in the complex PLs systems.

Supplementary Figure 10. TEM and DLS of the complex PLs systems.

Supplementary Figure 11. Viability of the Hela cells Cell.

Supplementary Figure 12. Photo-stability of molecule 3 in toluene.

Supplementary Figure 13. Structural characterization of compounds 1, 2 and 3.

Supplementary Table 1. Calculated energy of compounds 1, 2 and 3.

Supplementary Table 2. Photoluminescence quantum yield of compound 1 and 2 in DCM.

Supplementary Table 3. Photophysical properties of temperature dependence for compound 3.

Supplementary Table 4. Dihedral angles between donor and acceptor.

Supplementary Table 5. Pictorial representation of the natural transition orbitals.

Supplementary Table 6. Photoluminescence quantum yield of compound 3 in different solvents.

Supplementary Table 7. Wavelength and lifetime of 3-PLs-Chol.

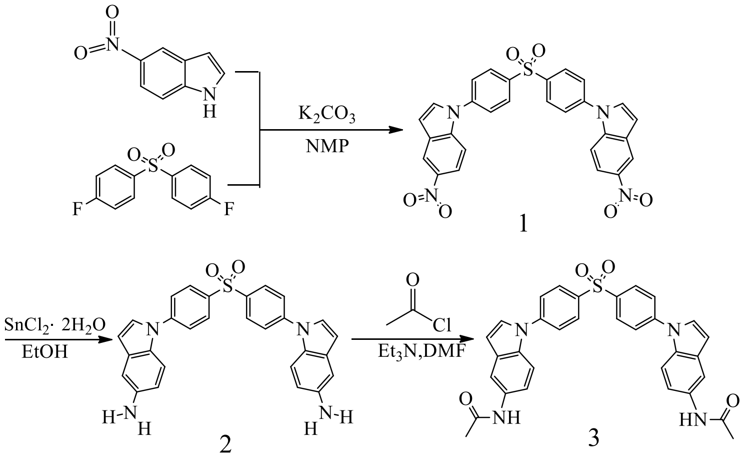

Supplementary Figure 1. Synthetic routes for compounds 1, 2 and 3.

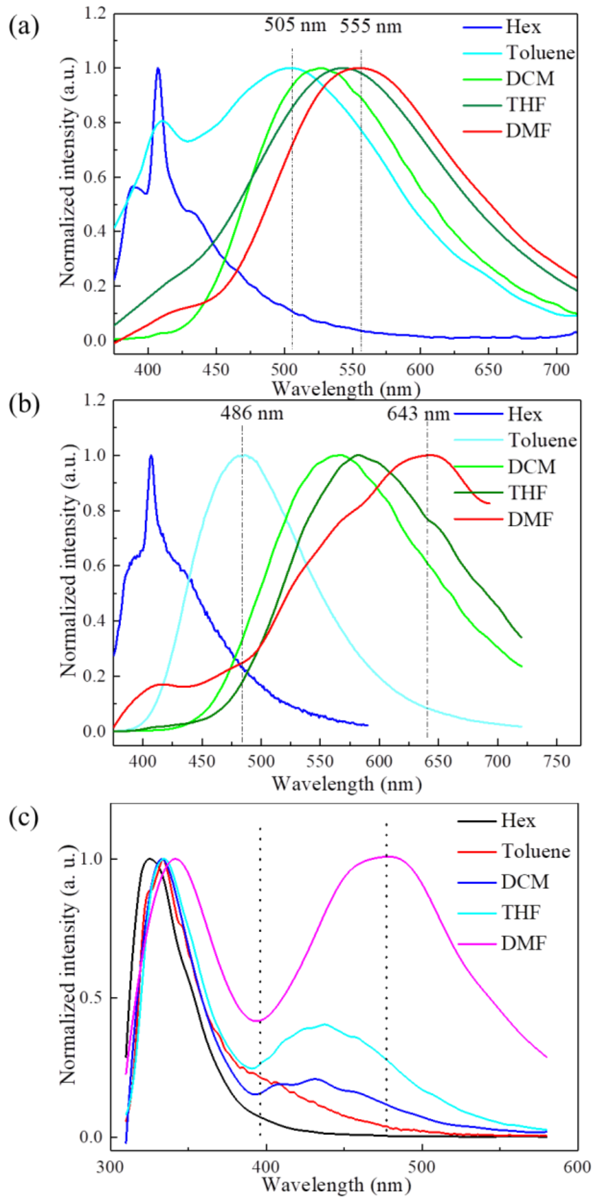

Supplementary Figure 2. Emission spectra in various solvents. (a) Compound 1, (b) Compound 2 and (c) Compound 3. The sharp peak at ~410 nm in (a) and (b) is assigned to the solvent Raman signal.

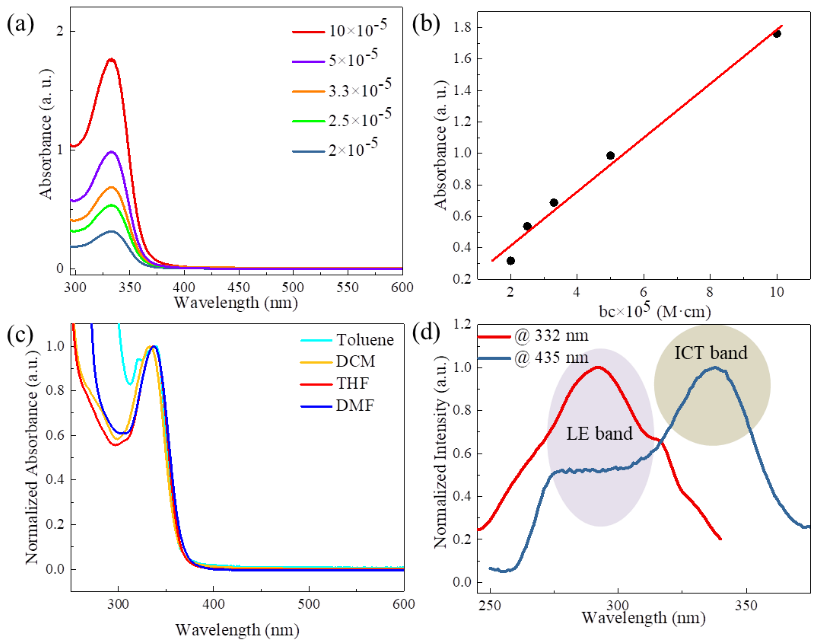

Supplementary Figure 3. Absorption and Excitation spectra of 3. (a) UV-Vis spectra of compound 3 in DCM at different concentrations. (b) Linear-fitting curve of the absorbance against bc ×10^5 (b: optical length, cm; c: concentraction of compound 3 in DCM, M). (c) Normalized UV-Vis spectra of compound 3 in different solvents. (d) Excitation spectra (λem = 332 nm and 435 nm) in DCM.

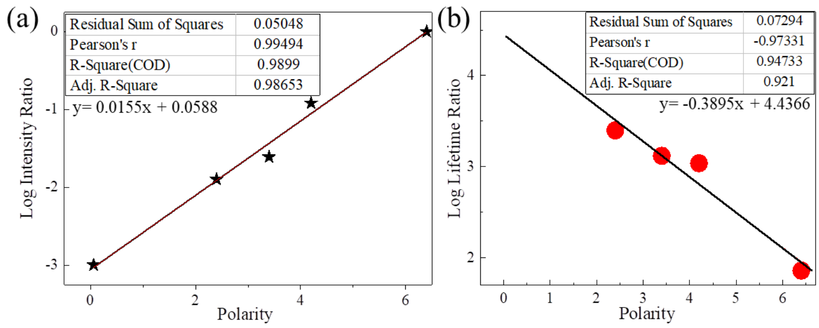

Supplementary Figure 4. Linear-fitting Correlation. The Log value of (a) wavelength and (b) lifetime ratio (TADF to FL) with polarity.

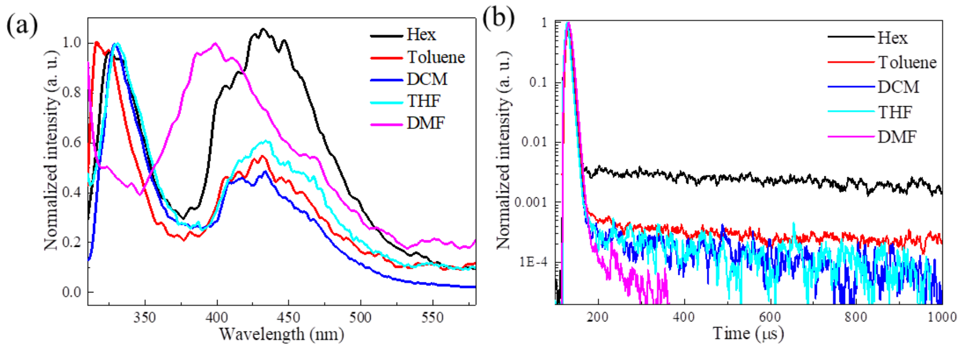

Supplementary Figure 5. Emission and lifetime in different solvents at 77K. (a) Emission spectra and (b) Phosphorescence decay.

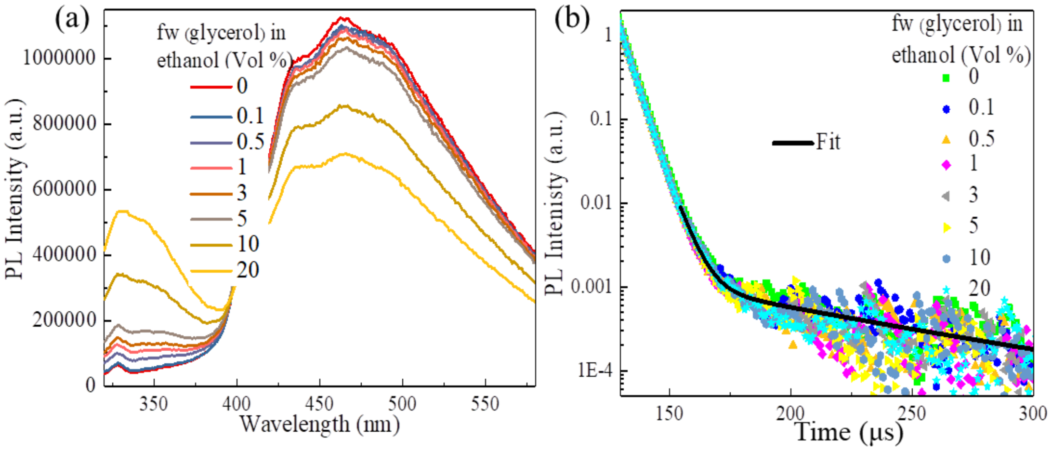

Supplementary Figure 6. Photophysical properties in ethanol with different glycterol concentrations. (a) Steady state PL and (b) Transient PL decay spectra.

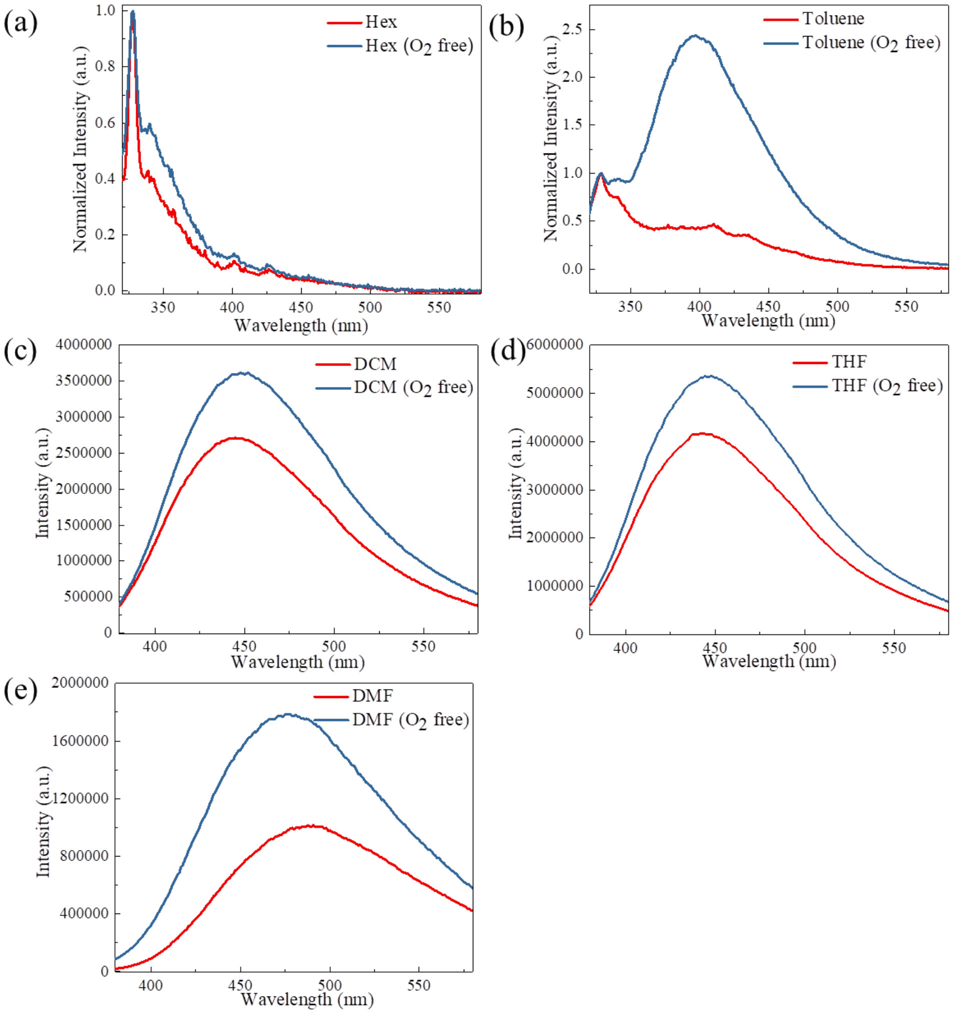

Supplementary Figure 7. Emission spectra in oxygen free and saturated air condition. (a) Hex (b) Toluene (c) DCM (d) THF and (e) DMF.

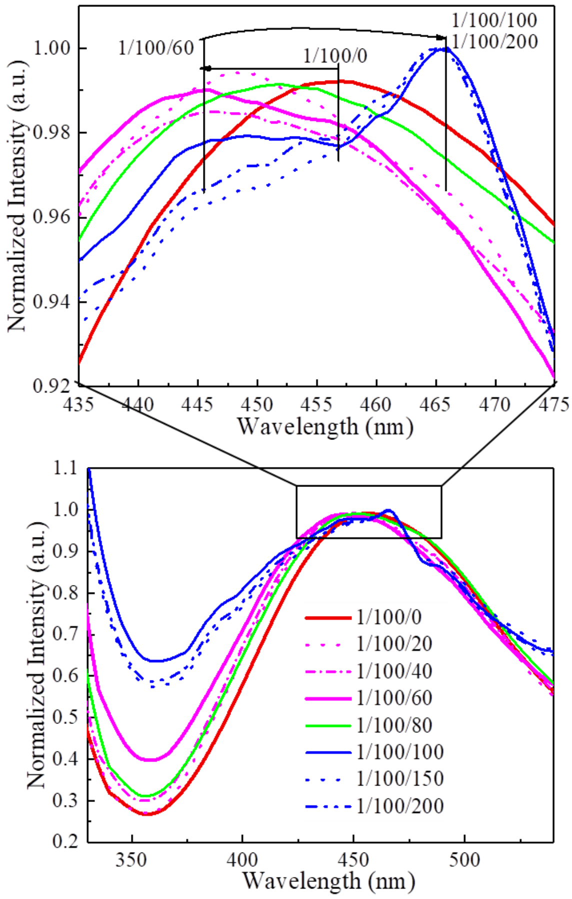

Supplementary Figure 8. Emission spectra of 3 in the complex PLs systems.

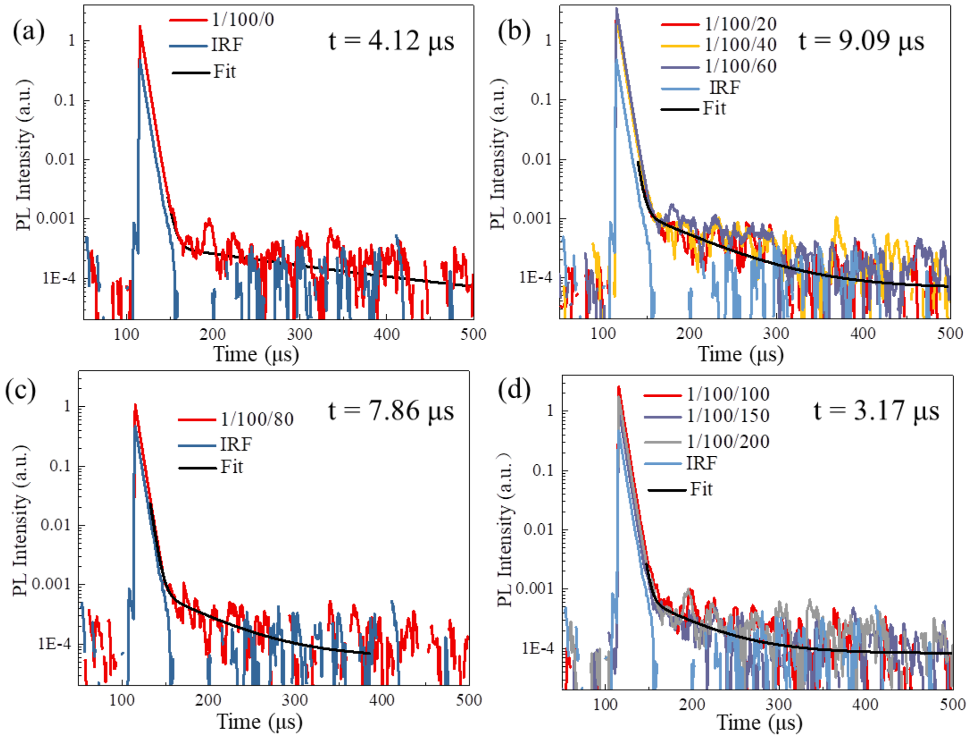

Supplementary Figure 9. Photoluminescence lifetime of 3 in the complex PLs systems.

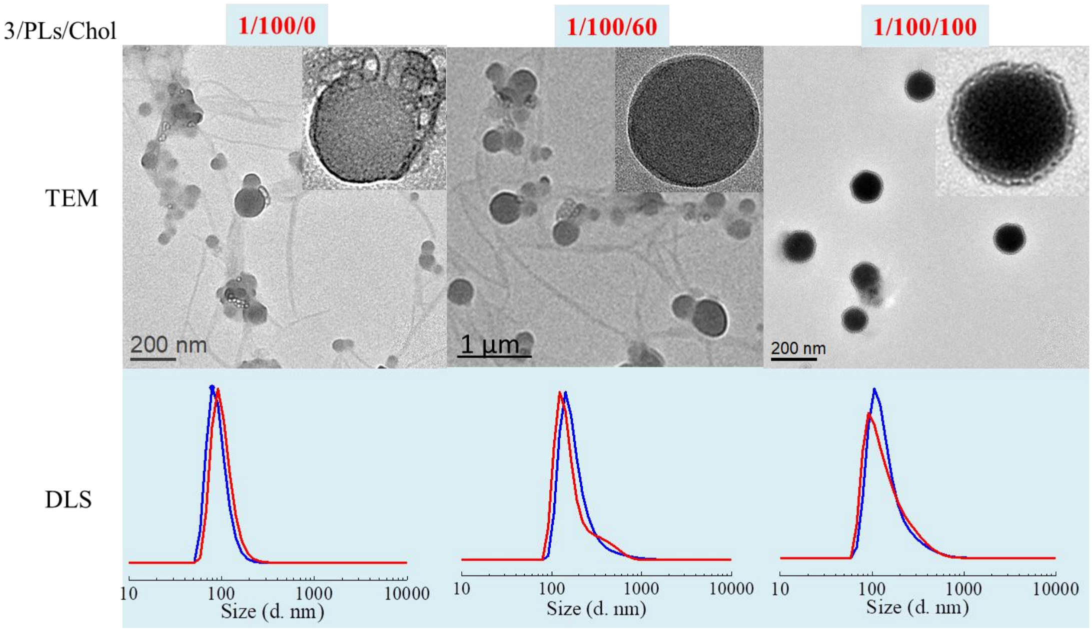

Supplementary Figure 10. TEM and DLS of the complex PLs systems.

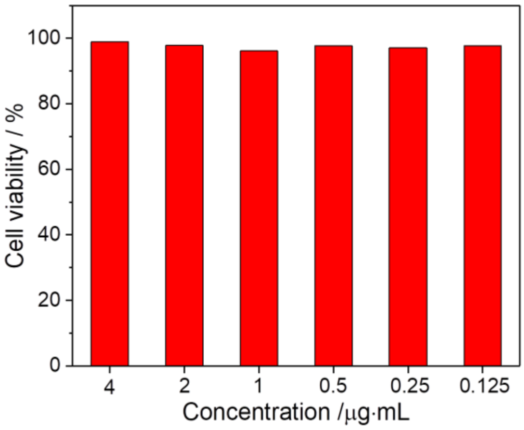

Supplementary Figure 11. Viability of the Hela cells Cell. The cells incubated with different concentration compound 3 via CCK-8 assay.

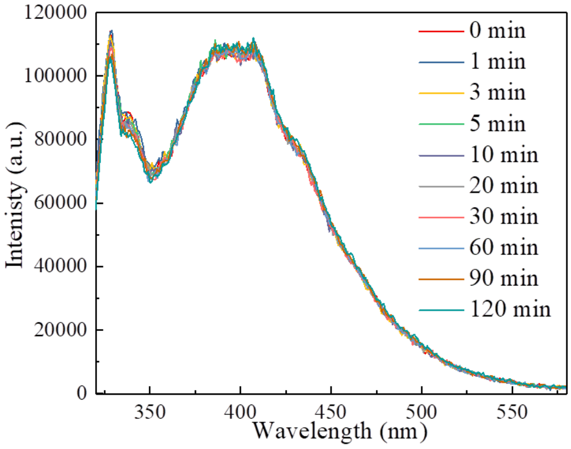

Supplementary Figure 12. Photo-stability of molecule 3 in toluene. (under 300 nm light within 120 min)

(a)

1

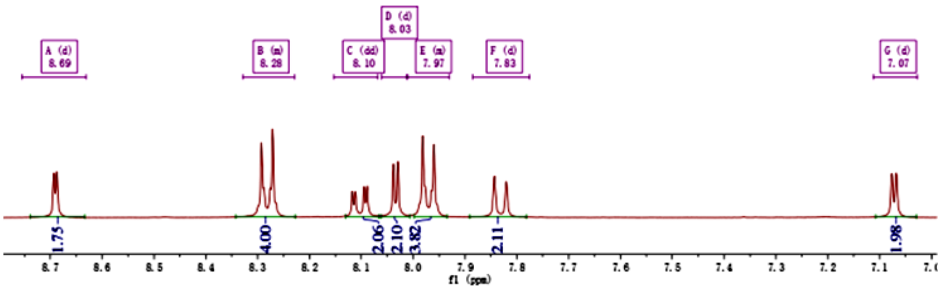

A (d) 8.69
B (m) 8.28
C (dd) 8.10
D (d) 8.03
E (m) 7.97
F (d) 7.83
G (d) 7.07
f1 (ppm)

2

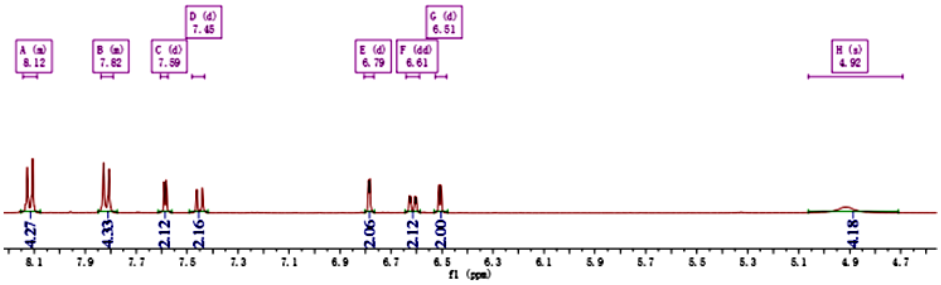

A (m) 8.12
B (m) 7.82
C (d) 7.59
D (d) 7.45
E (d) 6.79
F (dd) 6.61
G (d) 6.51
H (s) 4.92
f1 (ppm)

3

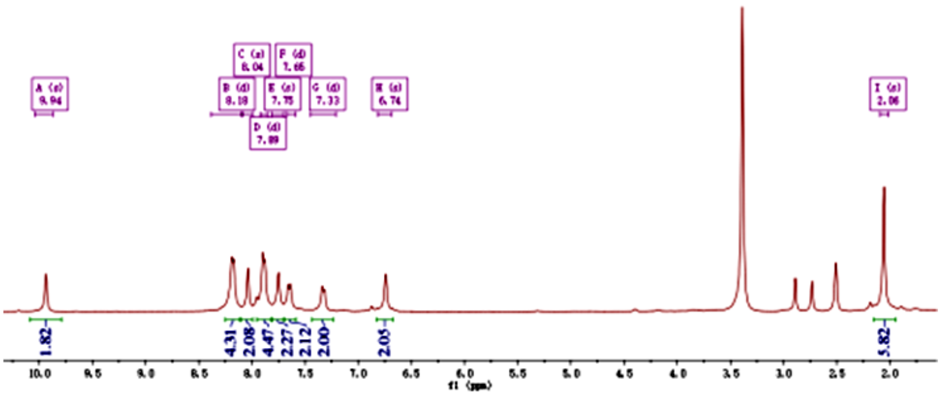

A (s) 9.94
B (d) 8.18
C (s) 8.04
D (d) 7.89
E (s) 7.75
F (d) 7.65
G (d) 7.33
H (s) 6.74
I (s) 2.06
f1 (ppm)

(b) 1

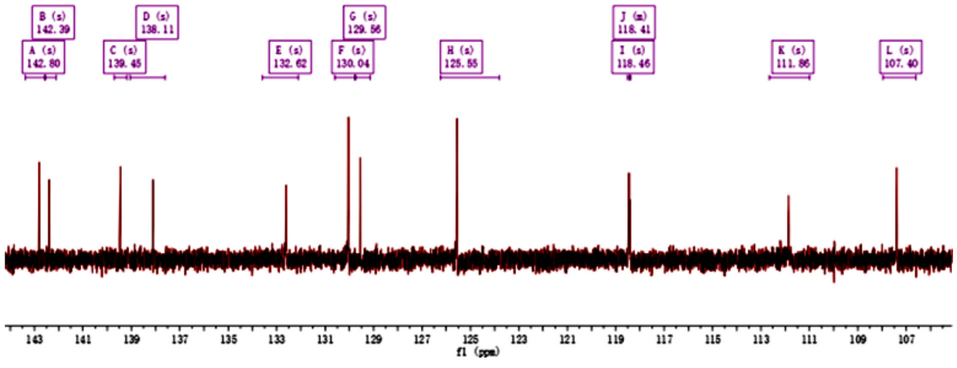
B (s) 142.39
A (s) 142.80
C (s) 139.45
D (s) 138.11
E (s) 132.62
G (s) 129.56
F (s) 130.04
H (s) 125.55
J (m) 118.41
I (s) 118.46
K (s) 111.86
L (s) 107.40
143 141 139 137 135 133 131 129 127 125 123 121 119 117 115 113 111 109 107
f1 (ppm)

2

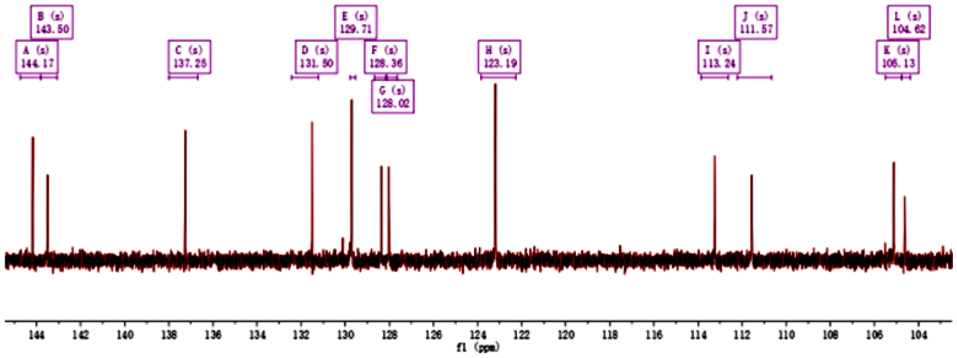
B (s) 143.50
A (s) 144.17
C (s) 137.25
D (s) 131.50
E (s) 129.71
F (s) 128.36
G (s) 128.02
H (s) 123.19
I (s) 113.24
J (s) 111.57
L (s) 104.62
K (s) 105.13
144 142 140 138 136 134 132 130 128 126 124 122 120 118 116 114 112 110 108 106 104
f1 (ppm)

3

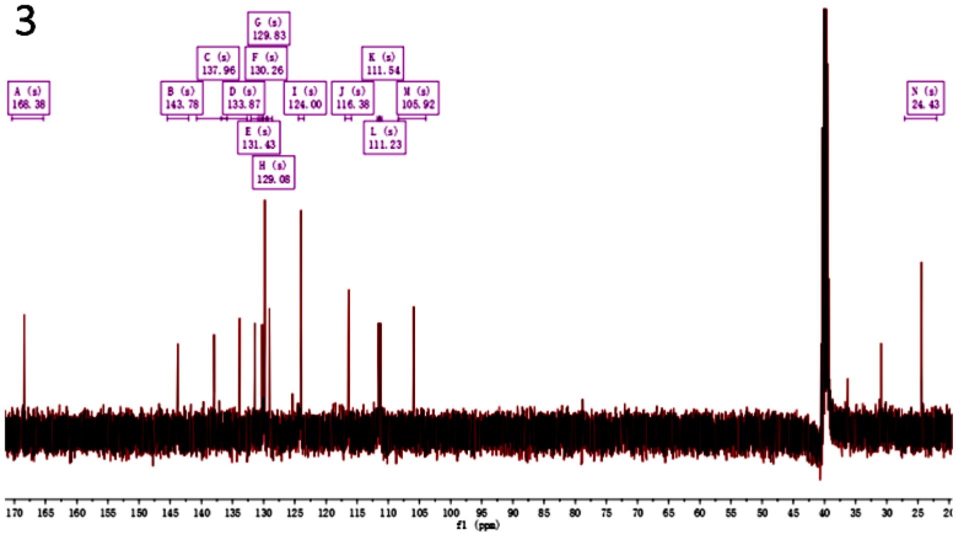
A (s) 168.38
B (s) 143.78
C (s) 137.96
D (s) 133.87
E (s) 131.43
G (s) 129.83
F (s) 130.26
H (s) 129.08
I (s) 124.00
J (s) 116.38
K (s) 111.54
L (s) 111.23
M (s) 105.92
N (s) 24.43
170 165 160 155 150 145 140 135 130 125 120 115 110 105 100 95 90 85 80 75 70 65 60 55 50 45 40 35 30 25 20
f1 (ppm)

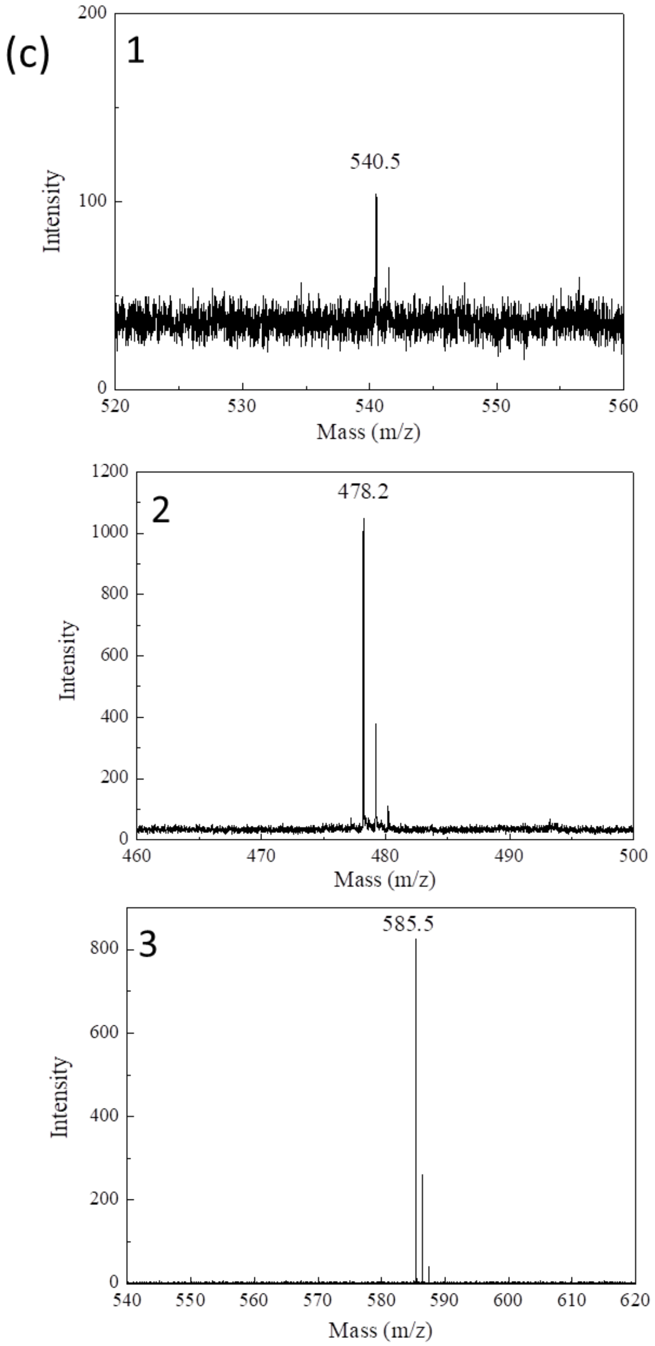

(c)
1
540.5
Intensity
Mass (m/z)
2
478.2
Intensity
Mass (m/z)
3
585.5
Intensity
Mass (m/z)

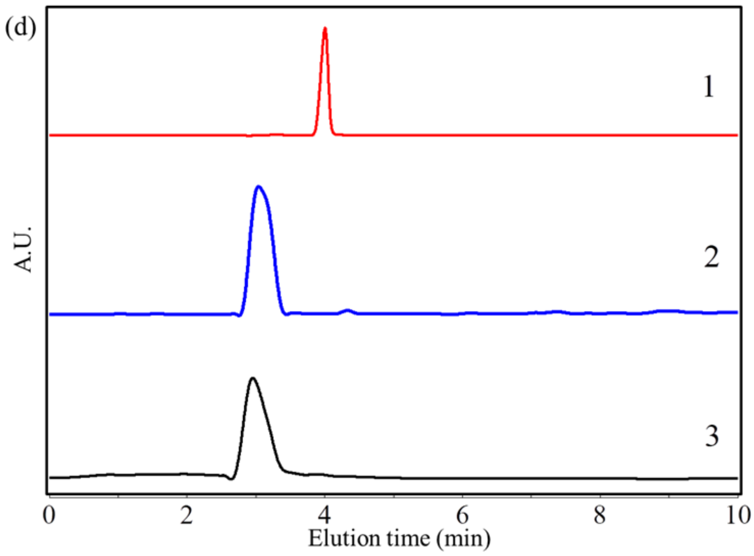

Supplementary Figure 13. Structural characterization of compounds 1, 2 and 3. (a) ^{1}H NMR spectrum, (b) ^{13}C NMR spectrum, (c) Maldi-tof mass spectrum and (d) HPLC.

Supplementary Table 1. Calculated energy of compounds 1, 2 and 3. Excitation energy and singlet–triplet energy gaps computed within vertical approximation (in eV).

	$E(S_0\text{-}S_1)$	$E(S_0\text{-}T_1)$	$E(S_0\text{-}T_2)$	$\Delta E(S_1T_1)$	$\Delta E(S_1T_2)$
1	2.73 (0.04)[a]	2.38	2.38	0.35	0.35
2	2.78 (0.26)[a]	2.61	2.68	0.17	0.10
3	3.09 (0.40)[a]	2.86	2.97	0.23(0.173)[b]	0.12(0.10)[b]

[a] oscillator strength values in parentheses.

[b] experimental value.

Supplementary Table 2. Photoluminescence quantum yield of compound 1 and 2 in DCM.

compound	PLQY
1	0.06
2	0.17

Supplementary Table 3. Photophysical properties of temperature dependence for compound 3. Compound 3 doped DPEPO film.

T, K	τ_p, ns	k_p, s^{-1}	τ_d, µs	k_d, s^{-1}	I_d/I_p
300	22	4.55×10^7	29	3.45×10^4	0.2
290	22	4.55×10^7	37	2.70×10^4	0.18
280	21	4.76×10^7	49	2.04×10^4	0.15
270	20	5.00×10^7	65	1.54×10^4	0.12
260	20	5.00×10^7	87	1.15×10^4	0.1
250	19	5.26×10^7	118	8.47×10^3	0.06
240	19	5.26×10^7	162	6.17×10^3	0.04
230	18	5.56×10^7	226	4.42×10^3	0.02
220	18	5.56×10^7	318	3.14×10^3	0.012
210	17	5.88×10^7	456	2.19×10^3	0.008
200	17	5.88×10^7	665	1.50×10^3	0.005

Supplementary Table 4. Dihedral angles between donor and acceptor. Dihedral angles between the indole-based donor and the phenylsulfone acceptor moieties for 1~3 in the ground singlet (S_0) and first excited S_1 and T_1 states.

	DA(a)[DA(b)] S_0	DA(a)[DA(b)] S_1	DA(a)[DA(b)] T_1
1	45° [45°]	45° [45°]	47° [45°]
2	41° [41°]	46° [90°]	46° [85°]
3	41° [40°]	48° [93°]	38° [38°]

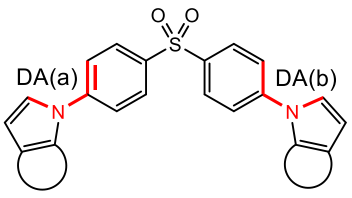

Supplementary Table 5. Pictorial representation of the natural transition orbitals. Describing the S_1 /T_1/T_2 → S_0 process for the molecule. The weight of the hole–electron contribution to the excitation is also included.

	S_1		T_1		T_2	
1	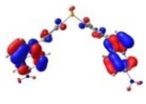	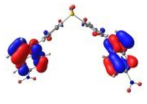	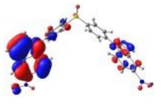	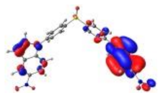	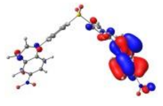	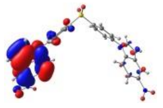
weight	ν=0.734	ν=0.261	ν=0.593	ν=0.404	ν=0.580	ν=0.418
	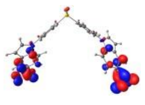	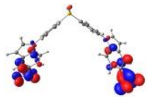	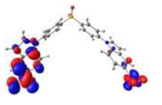	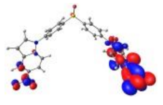	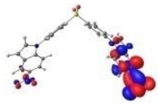	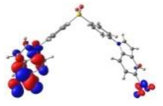
2	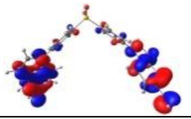		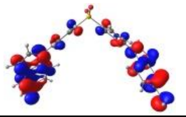		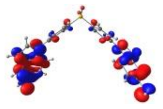	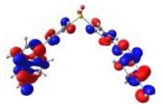
weight	ν=0.996		ν=0.950		ν=0.937	ν=0.059
	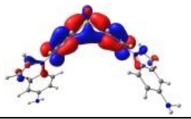		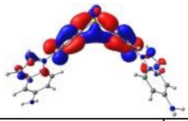		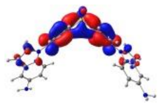	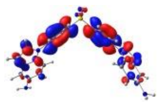
3	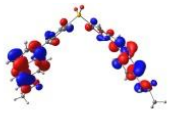		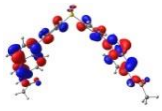	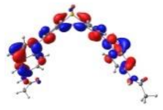	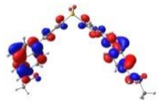	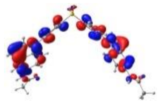
weight	ν=0.993		ν=0.923	ν=0.061	ν=0.876	ν=0.109
	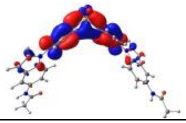		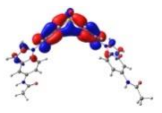	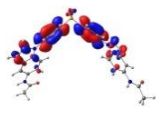	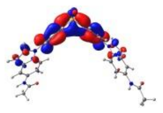	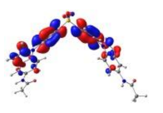

Supplementary Table 6. Photoluminescence quantum yield of compound 3 in different solvents.

solvents	Hexane	Toluene	DCM	THF	DMF
PLQY (saturated air)	0.01	0.12	0.38	0.42	0.12
PLQY (O2 free)	0.05	0.33	0.44	0.48	0.28

Supplementary Table 7. Wavelength and lifetime of 3-PLs-Chol.

Mass ratio of 3-PLs-Chol	Wavelength of FL (nm)	Lifetime of FL (ns)	Wavelength of TADF (nm)	Lifetime of TADF (μs)
1-100-0	332	22	457	4.12
1-100-20	332	22	448	9.09
1-100-40	332	22	446	9.09
1-100-60	332	22	445	9.09
1-100-80	332	22	452	7.86
1-100-100	332	22	466	3.17
1-100-150	332	22	466	3.17
1-100-200	332	22	466	3.17
